# Deep Learning-Based Evaluation of Postural Control Impairments Caused by Stroke Under Altered Sensory Conditions

**DOI:** 10.3390/biomimetics10090586

**Published:** 2025-09-03

**Authors:** Armin Najipour, Siamak Khorramymehr, Mehdi Razeghi, Kamran Hassani

**Affiliations:** Department of Biomedical Engineering, College of Medical Science and Technologises, Tehran Science and Research Branch, Islamic Azad University, Tehran 1477893855, Iran

**Keywords:** CNN, sensory disorders, stroke, deep learning

## Abstract

Accurate and timely detection of postural control impairments in stroke patients is crucial for effective rehabilitation and fall prevention. Traditional clinical assessments often rely on qualitative observation and handcrafted features, which may fail to capture the nonlinear and uncertain nature of postural deficits. This study addresses these limitations by introducing a hybrid deep learning framework that integrates Convolutional Neural Networks (CNNs) with Type-2 fuzzy logic activation to robustly classify sensory dysfunction under altered balance conditions. Using an EquiTest-derived dataset of 8316 labeled samples from 700 participants across six standardized sensory manipulation scenarios, the proposed method achieved 97% accuracy, 96% precision, 97% sensitivity, and 96% specificity, outperforming conventional CNN and other baseline classifiers. The approach demonstrated resilience to measurement noise down to 1 dB SNR, confirming its robustness in realistic clinical environments. These results suggest that the proposed system can serve as a practical, non-invasive tool for clinical diagnosis and personalized rehabilitation planning, supporting data-driven decision-making in stroke care.

## 1. Introduction

Postural control is a complex neurophysiological process that enables individuals to maintain balance and orientation during static and dynamic activities. Accurate assessment of postural stability is essential for identifying neurological disorders such as stroke, which often disrupt multisensory integration and motor coordination. In recent years, there has been growing interest in developing computational methods to analyze balance impairments using intelligent systems, particularly in stroke rehabilitation contexts [1,2,3].

Traditional clinical assessments rely on qualitative observations or handcrafted features extracted from balance signals [4]. However, these approaches often fail to capture the nonlinear and uncertain nature of sensory deficits [5]. Deep learning has emerged as a powerful tool for analyzing biomedical signals due to its ability to learn complex patterns directly from raw data without manual feature engineering. Convolutional Neural Networks (CNNs), in particular, have shown promise in time-series analysis tasks, including those involving physiological signals [6,7].

Despite the success of CNNs, one of their limitations lies in their sensitivity to noisy or ambiguous input data, especially when dealing with human sensorimotor systems affected by disease [8,9]. To address this, hybrid approaches that integrate fuzzy logic—especially type-2 fuzzy systems—have been proposed to enhance model robustness in the presence of uncertainty and environmental noise. Type-2 fuzzy sets can model higher-order vagueness, making them well-suited for real-world biomedical applications [10,11,12].

In this paper, we propose a novel deep learning-based model that combines CNNs with type-2 fuzzy activation functions to classify postural control impairments in stroke patients under altered sensory conditions. The model is evaluated on a dataset collected using the EquiTest platform, which simulates six different sensory balance conditions. We demonstrate that the hybrid model not only achieves high classification accuracy but also remains robust in noisy environments, making it a promising tool for automated balance assessment. The proposed hybrid model in this study offers several key advantages:

A: Achieving rapid and accurate classification of postural signals under different sensory conditions using deep learning techniques, enabling real-time or near-real-time assessment in clinical settings.
B: Demonstrating the advantages of deep learning-based models over traditional statistical approaches in analyzing complex physiological signals with high-dimensional and nonlinear characteristics.
C: Eliminating the need for manual feature selection by leveraging automated feature extraction capabilities of convolutional neural networks.
D: Evaluating the performance of the proposed hybrid model (CNN + Type-2 Fuzzy Logic) under diverse uncertainties including sensor noise and inter-subject variability, which commonly occur in real-world clinical data.
E: Providing a practical modeling approach for the classification of balance impairments based on raw or minimally pre-processed center of pressure (CoP) signals, thereby simplifying the processing pipeline.
F: Integrating a deep CNN with Type-2 fuzzy logic to enhance robustness and algorithmic stability in postural classification tasks, particularly under noisy or uncertain measurement conditions.

The remainder of this paper is organized as follows: Section 2 reviews related works on machine learning-based postural assessment. Section 3 details the dataset and proposed hybrid model. Section 4 presents the experimental results. Section 5 discusses the findings, and Section 6 concludes the study with directions for future research.

## 2. Related Works

This section presents a comprehensive review of previous studies relevant to postural control assessment and stroke rehabilitation. Given the interdisciplinary nature of this work—spanning biomedical signal analysis, clinical evaluation, and intelligent systems—we have divided the review into two main categories: Section 2.1 summarizes key clinical studies that have investigated balance impairment, rehabilitation protocols, and sensory manipulation techniques. These studies are presented in a comparative format to highlight their methodologies, datasets, and focus areas. Section 2.2 focuses on recent advances in artificial intelligence (AI) and machine learning techniques that have been applied to postural analysis. In particular, it emphasizes the use of deep learning models such as convolutional neural networks (CNNs) and hybrid systems involving fuzzy logic for robust classification under uncertainty. Together, these subsections provide a dual perspective on the current landscape: one grounded in clinical practice and the other in computational innovation.

### 2.1. Clinical Studies and Prior Applications

Numerous studies have investigated indices that assess the three elements of human body balance (stability, dynamic stability, weight bearing) in an upright position under diverse conditions and ailments. A subset of these studies has examined sabermetric indices concerning the stability of the human body’s balance system in an upright position, presuming linearity of body oscillations by assessing the trajectory of the center of pressure. Chiari et al. [13] examined dynamic indices of postural control and presented a comprehensive reference of 55 indices in both anteroposterior and lateral directions within a plane and across three temporal domains. They have examined the correlation between each of the 29 frequencies and random parameters of the indicators with attributes such as age, height, weight, location, foot position, and type of support surface. Norris et al. [14] assessed three cohorts—young, elderly, and unstable elderly individuals—utilizing posturographic indices to examine fall risk. They determined that conventional linear dynamic indices offer insufficient insight into the system’s transient characteristics. Li et al. [15] examined postural control by analyzing the contributions of small and large sensory afferents in individuals with peripheral neuropathy. This study aimed to elucidate the role of sensory feedback in postural control and examine how this relationship is generally influenced by prevalent forms of peripheral neuropathy (PN). A thorough examination of the scientific literature via MEDLINE was performed, and pertinent information was consolidated. Evidence indicates that proprioceptive feedback, primarily facilitated by larger type I afferents, is crucial for postural regulation. Evidence indicates that tactile feedback through smaller type II afferents is crucial for maintaining balance. Numerous types of peripheral neuropathy frequently led to persistent tactile desensitization in the plantar surfaces of the feet. This study presents a model to elucidate the relationship between the stability and feedback of smaller type II afferents and the feedback of larger type I afferents, which may be compromised by peripheral neuropathy, to assist in the formulation of pertinent rehabilitation strategies. Kiwanara et al. [16] proposed a model for human postural control based on dissociated sensory systems. This study elucidates the reasons older adults experience dizziness and their preference for strategies beyond the ankle strategy. Richmond et al. [17] offered a comprehensive analysis of compromised postural control in individuals with multiple sclerosis and the related neural mechanisms. This study evaluated postural control performance using global and directional time-to-boundary metrics across four altered sensory conditions (eyes open/closed, firm/foam) in twenty-nine neurotypical individuals and twenty-seven persons with multiple sclerosis (PwMS). The postural outcomes were evaluated using an analysis of variance in both the MS and control groups. Postural performance correlated with diffusion tensor magnetic resonance imaging assessments of CSP microstructural integrity. Individuals with multiple sclerosis exhibited inferior anterior-posterior posture relative to their non-neural counterparts and demonstrated diminished microstructural integrity compared to non-neural adults. Da Oliveira et al. [18] assessed the immediate impact of core stability and sensorimotor training on postural control in both seated and standing positions among young adults. This study involved 39 participants with a mean age of 23 years, who were randomly allocated to two groups: (a) core stability training and (b) sensorimotor training. The participants’ posture was evaluated pre- and post-training in both seated and one-legged standing positions utilizing a force platform. No notable differences were detected in any of the postural fluctuation variables between the two study cohorts. The interventions exhibited a generally small to moderate effect size. The findings indicated that acute intervention involving core stability and sensorimotor training did not significantly impact the sitting and standing postures of young adults. Plereti et al. [19] conducted a systematic review to assess the utilization of unsupervised machine learning in research on autism spectrum disorder (ASD) for predictive purposes. This study analyzed 43 unsupervised machine learning algorithms in ASD, including k-nearest neighbor clustering, hierarchical clustering, model-based clustering, and self-organizing maps. This review aims to survey contemporary applications of unsupervised machine learning in ASD research and to elucidate the types of inquiries that these methods can address. Deniz Tuncer et al. [20] examined postural control in children exhibiting heightened femoral deviation to regulate their posture. This study examined sixteen children with Iron Deficiency Anemia (IF A) aged 10–15 years alongside a control group of sixteen age-matched typically developing children. Postural stability (PS), limits of stability (LoS), and the modified clinical test of sensory integration of balance (mCTSIB) were employed to evaluate postural control via the balance system (BBS) and the balance error scoring system (BESS). Visual observation of instability was conducted in three standing positions across six different scenarios for all cases. An independent *t*-test in SPSS v.20 was employed to compare groups based on the characteristics of data distribution. The findings indicated substantial disparities among groups regarding the overall and anterior/posterior stability index in PS, all LoS parameters, and mCTSIB. The findings of this study indicated that postural stability and balance are compromised in healthy children with IFA. Xu et al. [21] assessed posture utilizing motion sensors. The proposed solution comprises flexible sensors that monitor all spinal movements, capable of detecting postural deviations and assisting in posture retraining. Photogrammetry is employed to identify body parts and quantify their asymmetry. This study involved twelve experiments to assess the system’s repeatability and evaluate the asymmetry of the chosen limb. Consequently, all alterations ranged from 0 to 1 cm in 9 students, demonstrating the efficacy of the proposed method in this study. The findings from conventional methods indicated that both approaches demonstrate favorable outcomes for the diagnosis and treatment of ailments resulting from poor posture. The research indicates that numerous studies have been undertaken regarding body balance indices. Nonetheless, these studies possess limitations. Cherstvy et al. [22] investigated diffusion processes with exponential and logarithmic time-dependent diffusion coefficients. The authors identified significant differences in diffusion behavior, non-ergodicity, and aging phenomena between particles with mass (such as Brownian particles) and particles without mass (such as photons). This study showed that the time dependence of the diffusion coefficient has a significant impact on the dynamic behavior of various physical systems. Novikov et al. [23] reviewed the use of magnetic resonance spectroscopy (MRS) in cancer diagnosis and management. This article shows that MRS can be useful in the diagnosis, localization, staging, assessment of tumor invasion, and evaluation of tumor response to therapy in various cancers including brain, breast, prostate, and liver. Table 1 lists the review studies along with the methodology used.

The primary challenge in prior research is the application of statistical methods alongside significant and insignificant indicators. These methods rely on individuals’ self-reports, rendering the ultimate diagnostic accuracy susceptible to error. The second challenge in research is the utilization of manual (traditional) feature selection and extraction techniques. Although these methods are precise, their utilization is suboptimal. These methods do not ensure the optimality of the feature vector for the classifier. Furthermore, it can enhance the algorithm’s computational efficiency. The proposed approach, integrating deep learning techniques with type 2 fuzzy networks, addresses the previously mentioned challenges and enables precise prediction of the conditions of healthy individuals and stroke patients under simulated sensory environments.

### 2.2. AI-Based Approaches for Postural Assessment

Recent advances in artificial intelligence (AI) and machine learning (ML) have provided powerful tools for automated and objective assessment of postural control impairments. These computational approaches have demonstrated strong capabilities in extracting complex patterns from physiological signals, often surpassing traditional statistical methods. Based on the literature, AI-based methods for postural assessment can be categorized into several main groups:

#### 2.2.1. CNN-Based Approaches

Convolutional Neural Networks (CNNs) have been extensively employed for analyzing balance-related time-series data due to their ability to automatically extract spatial and temporal features from raw inputs. For example, CNNs have been applied to force platform signals to distinguish healthy individuals from those with balance disorders, showing high classification accuracy without manual feature engineering [24]. Wearable-sensor-based CNN models have also been developed for continuous postural monitoring and early detection of instability [25]. However, despite their strong representational power, pure CNN architectures can be sensitive to sensor noise, variations in data acquisition conditions, and inter-subject variability, limiting their robustness in uncontrolled clinical environments.

#### 2.2.2. Hybrid CNN-RNN Models

To address temporal dependencies in postural sway and gait data, researchers have combined CNN layers for feature extraction with Recurrent Neural Networks (RNNs), particularly Long Short-Term Memory (LSTM) units or Gated Recurrent Units (GRUs), for sequence modeling. Such hybrid frameworks have been successful in recognizing complex movement patterns, detecting falls, and monitoring rehabilitation progress in real time [25,26]. These models are especially beneficial when the temporal evolution of balance signals carries diagnostic value. Nevertheless, their performance can degrade in the presence of high levels of measurement noise or when deployed across heterogeneous populations without domain adaptation.

#### 2.2.3. Fuzzy Logic and Type-1 Systems

Fuzzy inference systems, particularly Type-1 fuzzy logic, have been utilized to handle uncertainty and imprecision in postural measurements [27,28]. These systems incorporate expert-defined membership functions and rule bases to mimic human reasoning, making them interpretable and adaptable. In rehabilitation settings, fuzzy logic has been used for decision-making, adaptive feedback, and classification of balance impairments. However, Type-1 fuzzy logic systems are constrained in their ability to manage higher-order uncertainty and ambiguity, which often arise in clinical data with varying noise levels and individual differences.

#### 2.2.4. Hybrid Deep Fuzzy Models

More recently, researchers have explored hybrid architectures that integrate deep learning’s feature learning capabilities with the uncertainty modeling strengths of fuzzy logic. Deep fuzzy CNNs have been applied to tasks such as sleep stage classification [29], gait recognition [30], and tremor detection in Parkinson’s disease [31], demonstrating improved classification stability in noisy or ambiguous conditions. Among fuzzy systems, Type-2 fuzzy logic offers enhanced capacity to model uncertainty by incorporating a footprint of membership functions, making it particularly relevant for biomedical signal analysis where variability is high. Despite these advances, few studies have specifically applied Type-2 fuzzy activation functions within CNN frameworks for postural control assessment under multi-sensory conditions.

#### 2.2.5. Gap and Motivation for This Study

While the literature demonstrates diverse and promising applications of AI for balance assessment, most prior works are limited to unimodal datasets, simplified sensory conditions, or Type-1 fuzzy reasoning. Furthermore, few models have been explicitly optimized for robustness against both sensor noise and inter-subject variability—two critical factors in real-world clinical deployment. Our proposed approach addresses these gaps by combining CNN-based spatial feature extraction with a Single-Input Type-2 Fuzzy Rectifying Unit (SIT2-FRU) activation mechanism. This integration enhances robustness to noise, improves generalization across diverse populations, and maintains high accuracy under altered sensory conditions, making it particularly suitable for stroke-related postural assessment in clinical and rehabilitation contexts.

## 3. Methodology

### 3.1. Problem Statement

The core problem addressed in this study is the automated detection and classification of postural control impairments in stroke patients under altered sensory conditions. Impaired postural control is a major cause of disability and fall risk in stroke survivors, making its early and accurate detection a critical component of rehabilitation planning and outcome monitoring.

- Mathematical formulation: Let *X* ∈ *R^N^*^×*T*×*C*^ denote the multichannel time-series data of CoP signals, where *N* is the number of samples, *T* is the number of time steps, and *C* is the number of sensor channels. Each *Xi* corresponds to one of the six standardized Sensory Organization Test (SOT) scenarios, recorded via the EquiTest dynamic posturography system from either a healthy control or a stroke patient. The task is to learn a function: *f_θ_*:*Xi* ↦ *yi* where *yi* ∈ {0,1} represents the binary class label: 0 for healthy postural control and 1 for impaired postural control. The objective is to optimize the parameters *θ* of an Artificial Neural Network (ANN)-based model to minimize classification error while achieving high generalization across all sensory conditions and participant variability. Model performance is assessed using accuracy, precision, sensitivity, and specificity, with robustness further validated under Gaussian noise at SNR levels from 20 dB to 1 dB.

### 3.2. Overview of the Proposed Approach

In this study, we propose a novel hybrid classification model that integrates a deep convolutional neural network (CNN) with a Type-2 fuzzy inference system. This approach is designed to assess postural control signals under sensory perturbations and classify individuals based on their balance impairment levels. The input data consist of CoP signals collected from both healthy and stroke subjects under different sensory conditions. By leveraging the feature extraction capabilities of CNNs and the uncertainty modeling strength of Type-2 fuzzy logic, the proposed method aims to provide accurate and interpretable assessments suitable for clinical deployment.

Given its ability to classify postural control patterns with high accuracy and interpretability, the proposed hybrid model can be integrated into movement analysis laboratories, physiotherapy clinics, and neurological assessments to support objective evaluation of balance dysfunction.

It is descriptive as it illustrates the current situation, and it is correlational as it analyzes the evaluation of the posture control system in healthy individuals and stroke patients under sensory-stimulated conditions in the specified model. The proposed hybrid classification model is practical and has potential for real-world application in movement analysis labs, physiotherapy clinics, and neurological diagnostics. Healthy individuals and stroke patients were randomly selected for this purpose. The discussion focused on the design of a hybrid model for predicting sensory impairment using the proposed data mining algorithms, incorporating uncertainty through fuzzy logic. This study assessed the participants’ postural control system across six stages of the sensory organization test using dynamic dual force platforms and a dynamic cabin (EquiTest device—The EquiTest device was manufactured by NeuroCom International Inc., Clackamas, OR, USA.). Linear indices of state fluctuations and other relevant attributes are computed to create the feature vector for machine learning algorithms.

### 3.3. Data Collection

The dataset used in this study was originally collected in the Physiological Signal Processing Laboratory at the Faculty of Electrical Engineering, University of Tabriz. The data acquisition was part of an academic project aimed at demonstrating postural assessment techniques to biomedical engineering students and researchers. However, the dataset comprises real measurements obtained from healthy individuals and stroke patients under simulated sensory conditions. For the present research, a subset of these recordings was selected and reprocessed under ethical approval (IR.TBZ.REC.1400.150) to serve as input for the proposed classification model.

The dataset comprised 700 individuals, including 490 males and 210 females, with both healthy subjects and patients diagnosed with post-stroke postural impairments. Stroke patients were included based on clinical diagnosis and lesion duration, and varied in severity from moderate to severe, as documented in their medical reports.

The SOT was conducted using the EquiTest dynamic posturography system under six standard sensory manipulation conditions: a. Eyes open, fixed surface; b. Eyes closed, fixed surface; c. Sway-referenced vision; d. Eyes open, sway-referenced surface; e. Eyes closed, sway-referenced surface; f. Sway-referenced vision and surface.

Time-series data of postural sway were recorded for each participant in all six conditions. These data were used to form the labeled dataset for model training and testing. This study assessed the participants’ postural control system using dynamic dual force platforms and a dynamic cabin with the EquiTest device across six stages of the sensory organization test. Linear indices of postural fluctuations and additional frequently utilized characteristics were computed to create the feature vector. This research involved gathering data via a questionnaire evaluating the status control system of stroke patients, alongside a laboratory study utilizing the EquiTest device to record time series of postural fluctuations during various conditions of the sensory organization test. In total, 700 participants (490 male and 210 female) took part in this study. Each individual underwent multiple testing sessions across six sensory conditions defined by the SOT using the EquiTest system. For each condition, time-series data were recorded over several trials, and multiple samples were extracted from each participant’s performance. As a result, the dataset comprised 8316 labeled samples derived from the full set of trials and conditions. These samples were categorized into two groups: “normal” (healthy individuals) and “brain tumor” (stroke patients with postural control impairments), each contributing a proportion of the total sample pool.

### 3.4. Algorithms Employed in the Proposed Model

This subsection will review the mathematical foundations of the algorithms employed in the proposed architecture, encompassing deep convolutional networks and type 2 fuzzy functions.

#### 3.4.1. Deep Learning Networks

Deep learning networks constitute a subset of machine learning. Recent advancements in graphics hardware have garnered significant attention from researchers towards these networks. Deep learning networks can be employed in diverse applications, including medicine, agriculture, and engineering, necessitating high accuracy. Deep learning networks can be categorized into several sub-networks, including convolutional networks, recurrent neural networks, and adversarial networks. This research will examine the mathematical foundations of deep convolutional networks [32].

#### 3.4.2. Convolutional Neural Networks

**Figure 1:**
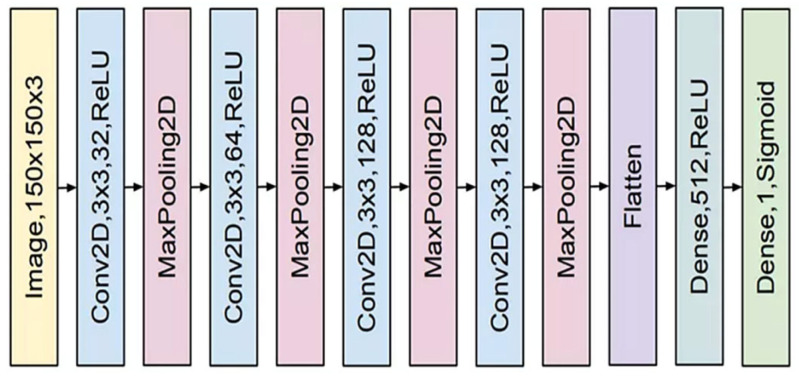
A general schematic illustration of a convolutional neural network (CNN) architecture, included to show the typical layer organization (convolutional, pooling, dropout, and dense layers) in deep learning models.

Convolutional neural networks are among the most significant deep learning architectures. This network was created by Mr. Hubble and Weissl in 1990, drawing inspiration from the human visual cortex. The primary application of these networks was the recognition of handwritten digits, yielding promising results. These networks were computationally impractical during that period due to insufficient graphics hardware and were subsequently discontinued. In recent years, the proliferation and enhancement of graphics processing units have revitalized the popularity of deep convolutional networks, which are now employed across diverse application domains. The resurgence of these networks can be assessed post-2012. The primary applications of convolutional neural networks are in object recognition, speech processing, and facial recognition. Convolutional networks, similar to neural networks, consist of layers of neurons equipped with weights and biases that can be optimized through learning. These networks consist of various blocks, as illustrated in Figure 1. The blue block in the illustration signifies the convolution layer featuring a nonlinear function. The red block signifies a pooling layer, while the green block denotes a fully connected layer. The convolutional layer is the fundamental component of the CNN. This layer, composed of various filters, can rotate on the input signal to generate the output feature map. The quantity of these filters is expressed as a power of 2, ranging from 32 to 4096. Augmenting the quantity of filters enhances the network’s capacity; however, this may lead to overfitting. The dimensions of the filters are specified as 3 × 3, 5 × 5, and 7 × 7, presented in a square format. The number of learnable parameters can be diminished by selecting smaller filters [33].

The step size, often referred to as the stride, acts as an additional parameter in the convolutional layer. It controls how far the filter moves during the convolution operation, thereby influencing how much the filtering effect persists across the input. In most convolutional networks, this parameter is two-dimensional (e.g., height and width). The operation of the convolutional layer, considering the stride, can be formally expressed as follows:

(1) $$S(i,j)=(X\times K)(i,j)=\sum_{\left(m=0\right)}^{\left(M-1\right)}\sum_{\left(n=0\right)}^{\left(N-1\right)}X(i+m,j+n)⊗K(m,n),$$

where “⊗” denotes the 2D convolution operation between the input region centered at position (*i*,*j*) and the kernel *K*. Here, *K*(*m*,*n*) is the kernel weight located at the *m*-th row and *n*-th column of the convolution filter, where the kernel is a small matrix of learnable parameters (e.g., 3 × 3) designed to extract specific features such as edges or textures. *X*(*i* + *m*,*j* + *n*) is the corresponding element in the input matrix, which represents the numerical form of the raw input data (e.g., pixel intensity values in an image) within the receptive field. *S*(*i*,*j*) represents the feature map value at position (*i*,*j*), indicating the activation strength of a specific learned feature after applying the convolution. This formulation describes how the convolution operation aggregates local regions of the input using the kernel weights to produce spatial feature representations. Activation functions are employed subsequent to the convolutional layer within the network. These functions induce nonlinearity within the network. Among the prevalent activation functions in convolutional networks are Rectified Linear Unit (ReLU) and Leaky ReLU. The ReLU function nullifies negative values in the network and transmits values exceeding zero to the output. This function is utilized by default in convolutional networks because of its simplicity and the enhancement of algorithm speed and network convergence [33]. The merging layer is an additional component in convolutional networks utilized to diminish the spatial dimensions of feature maps. This block lacks a training parameter and functions similarly to sampling, acting as a filter within the convolutional layer. The most prevalent filters are the max-merge and mean-merge layers. A preconfigured window measuring (3 × 3) is utilized for max-merge, which is traversed across the image to identify the maximum value, while the remaining values are set to zero. This layer reduces the feature dimensions. The ultimate layer of the convolutional network is the fully connected layer, wherein the classification of the selected or extracted features occurs. This layer is utilized in all classical neural networks. This layer transforms the chosen feature matrix into a vector and categorizes it into various designated classes. This layer computes the probability distribution of the output classes, which is articulated as follows:

(2) $$p_{i}=\frac{e^{x_{i}}}{\sum_{j=1}^{F}e^{x_{j}}}\;\mathrm{for}\;i=1,\;\ldots F,$$

In this equation, *p_i_* denotes the probability assigned to class *i*, *x_i_* is the raw score (logit) for that class, and *F* is the total number of classes, *x_j_* is the raw score (logit) for class *j*, used in the denominator for normalization; *e* is Euler’s number (the base of natural logarithms, approximately 2.718), and *j* is index variable used in the summation over all classes. The softmax function ensures that all *p_i_* values lie between 0 and 1, and their sum equals 1, making the output a valid probability distribution. The aforementioned layers are recognized as the primary layers in convolutional networks. Certainly, additional layers are employed in these networks, which will be elucidated subsequently. Additional frequently utilized layers comprise the random deletion layer and the batch normalization layer. The random deletion layer is employed to disregard the performance of certain neurons within the network. This layer inhibits the overfitting phenomenon in the network. The batch normalization layer is employed to standardize the data within the network. This layer diminishes internal covariance, consequently enhancing the training speed of the network. The subsequent relationship pertains to the efficacy of the batch normalization layer:

(3) $$\begin{array}{l} \mu_{B}^{\left(l-1\right)}=\frac{1}{n}\sum_{i=1}^{n}y_{i}^{\left(l-1\right)}\\ \sigma_{B}^{2(l-1)}=\frac{1}{n}\sum_{i=1}^{n}{\left(y_{i}^{\left(l-1\right)}-\mu_{B}\right)}^{2}\\ {\overset{\wedge }{y}}_{i}^{\left(l-1\right)}=\frac{y_{i}^{\left(l-1\right)}-\mu_{B}}{\sqrt{\left(\sigma_{B}^{2(l-1)}+\varepsilon \right)}}\\ z_{i}^{\left(l\right)}=\gamma^{\left(l\right)}{\overset{\wedge }{y}}_{i}^{\left(l-i\right)}+\beta^{\left(l\right)} \end{array},$$

where *n* is the number of samples in the mini-batch, $y_{i}^{\left(l-1\right)}$ is the activation of the *i*-th sample in layer *l* − 1, $\mu_{B}^{\left(l-1\right)}$ and $\sigma_{B}^{2(l-1)}$ are the batch-wise mean and variance, ^(*l*)^ and ^(*l*)^ are learnable scale and shift parameters in layer *l*, and ε is a small constant added for numerical stability [32].

#### 3.4.3. Fuzzy Sets

Type-1 fuzzy sets, introduced by Professor Lotfi Zadeh, are defined by membership functions that assign to each element a precise degree of membership in the range [0, 1]. While effective for many applications, they may be limited when the membership function itself is subject to uncertainty. Professor Zadeh later introduced the concept of Type-2 fuzzy sets to address this limitation and enhance the capabilities of the Type-1 variant.

These sets, possessing a degree of membership, can improve the robustness and effectiveness of systems under various uncertainties, including measurement noise [33]. This capability can enhance the design of control systems and sequences of numerical and lexical elements in type 2 fuzzy sets. As detailed in the section on convolutional networks, activation functions constitute a critical component of these networks. These functions are crucial to the learning process and enhance the efficacy of deep learning networks. Notwithstanding the popularity of these functions, their principal drawback is the nonlinearity of the relationship between input and output. This may compromise the stability of the network. Conversely, by taking into account the degrees of freedom of type 2 fuzzy sets, convolutional networks can achieve enhanced stability. Consequently, these type 2 fuzzy sets may be utilized in place of the ReLU and Leaky ReLU activation functions. Consequently, the subsequent relationship may be regarded for these sets:

(4) $$f\left(\sigma ;\gamma \right)=\left\{\begin{array}{ll} P\sigma k(\sigma ), & \mathrm{if}\;\sigma >0\\ N\sigma (-\sigma ), & \mathrm{if}\;\sigma \leq 0 \end{array}\right.,$$

Accordingly, the parameters in this formula can be expressed as follows: *σ*: denotes the normalized input to the Single-Input Type-2 Fuzzy Rectifying Unit (SIT2-FRU), computed as *σ* = *K_f_*·*x*, where *K_f_* = 1/max (|*x*|). It ensures *σ* ∈ [−1, 1], matching the domain of the fuzzy membership functions centered at −1, 0, and +1, *k*(*σ*): is a function that determines the membership rate; it is probably a kernel function or a type 1 membership function (such as Gaussian, triangular, etc.); *N*: are the adjustment coefficients for the positive and negative parts, which may model asymmetry or uncertainty on both sides of the function. In Equation (4), the symbol *P* denotes a crisp singleton value used in the consequent part of the fuzzy rule base. It determines the slope of the activation function in the positive quadrant and is one of the key design parameters of the SIT2-FRU. This approach is based on the interval type-2 fuzzy logic framework proposed by Beke and Kumbasar (2019) [33], where the output response is shaped by the parameters *P*, *N*, and *α*. Additionally, the term *k*(*σ*), defined in a later equation, serves as a fuzzy gain function and appears in both positive and negative regions of the activation input. For *σ* > 0, the activation is computed as *P*·*σ*·*k*(*σ*), and for *σ* ≤ 0, it becomes *N*·*σ*·*k*(−*σ*). Therefore, the function *k* is not omitted but rather used symmetrically with proper sign adjustment to maintain the continuity and consistency of the fuzzy mapping.

In the SIT2-FRU, the input value *x* is scaled using a normalization factor to produce *σ*, which serves as the actual input to the fuzzy activation layer. This step guarantees boundedness of σ and ensures compatibility with the triangular IT2 fuzzy sets used for fuzzy inference. This function can actually be used as an activation function or uncertainty mapping function in type 2 fuzzy. In this context, the function *k* is articulated as follows:

(5) $$k\left(\sigma \right)=\frac{1}{2}\left(\frac{1}{\alpha +\sigma -\alpha \sigma }+\frac{-1+\alpha }{-1+\alpha \sigma }\right),$$

In the above formula, *σ* is the function input (e.g., the difference, error, or distance from the center of the membership function) and *α* is a tuning parameter (e.g., the degree of curvature of the function or the confidence coefficient). This function acts as a weight or kernel function on the previous function and adjusts the final shape of the fuzzy function response. Among its properties, it can be said that if *α* is constant, the function (*σ*) varies in a specific way with *σ*.

The mathematical derivatives represent the learning parameters. Accordingly, the update parameters are also determined based on the following relationship:

(6) $$\frac{𝜕L}{𝜕\gamma_{C}}=\sum_{j}\frac{𝜕L}{𝜕f_{c}(\sigma_{cj})}\frac{𝜕f_{c}(\sigma_{cj})}{𝜕\gamma_{c}},$$

In this formulation, *c* denotes the layer index, *j* denotes the observation element index, and *L* represents the loss function (objective function) of the deep network. In this context, *P_c_* and *N_c_* are the parameters that define the slope of the fuzzy activation function in the positive and negative regions, respectively. These slopes are adjusted during training using gradients backpropagated from deeper layers.

(7) $$\frac{𝜕f_{c}(\sigma_{c})}{𝜕a_{c}}=\left\{\begin{array}{l} \frac{P_{c}\sigma_{c}}{2}(\frac{1}{\alpha_{c}\sigma_{c}-1}+\frac{\sigma_{c}-1}{{\left(a_{c}+\sigma_{c}-\alpha_{c}\sigma_{c}\right)}^{2}}+\frac{\sigma_{c}(1-a_{c})}{{\left(a_{c}\sigma_{c}-1\right)}^{2}})\\ \mathrm{if}\;\sigma_{c}>0\\ -\frac{N_{c}\sigma_{c}}{2}(\frac{1}{\alpha_{c}\sigma_{c}+1}+\frac{\sigma_{c}+1}{{\left(a_{c}-\sigma_{c}+\alpha_{c}\sigma_{c}\right)}^{2}}+\frac{\sigma_{c}(1-a_{c})}{{\left(a_{c}\sigma_{c}+1\right)}^{2}}\\ \mathrm{if}\;\sigma_{c}\leq 0 \end{array}\right.,$$

In this equation, *σ_c_* denotes the normalized input to the *c*-th fuzzy activation unit. The parameters *α_c_*, *P*_c_, and *N_c_* are the learnable values for that unit, representing the footprint of uncertainty, the slope in the positive region, and the slope in the negative region, respectively. These parameters are updated during training for each channel. Therefore,

(8) $$\begin{array}{l} \frac{𝜕f_{c}(\sigma_{c})}{𝜕P_{C}}=\left\{\begin{array}{ll} \sigma_{c}k_{c}(\sigma_{c}), & \;\mathrm{if}\;\sigma_{c}>0\\ 0, & \;\mathrm{if}\;\sigma_{c}\leq 0\; \end{array}\right.\\ \frac{𝜕f_{c}(\sigma_{c})}{𝜕N_{C}}=\left\{\begin{array}{ll} 0, & \mathrm{if}\;\sigma_{c}>0\\ \sigma_{c}k_{c}(-\sigma_{c}), & \mathrm{if}\;\sigma_{c}\leq 0 \end{array}\right. \end{array}.$$

The rule for updating the parameters *γ* by the momentum method is:

(9) $$∆\gamma =\rho ∆\gamma +\xi \frac{𝜕L}{𝜕\gamma },$$

where *ρ* represents the momentum terms, *ξ* is the learning rate, and *γ* displays the parameter changes at each learning step.

In this study, the objective function used for training the deep learning network is the categorical cross-entropy loss, which is widely used for multi-class classification tasks. The explicit form of the loss function is defined as:

(10) $$L=-\sum_{i=1}^{N}\sum_{o=1}^{c}y_{io.}\log({\hat{y}}_{io}),$$

where *N* denotes the total number of training samples, *C* denotes the total number of classes, *y_io_* ∈ {0,1} indicates whether sample *i* belongs to class *o*, ${\hat{y}}_{io}$ ∈ [0, 1] is the predicted probability (output of the softmax layer) that sample *i* belongs to class *o*. This loss function measures the dissimilarity between the true class distribution and the predicted class distribution. It is differentiable and suitable for backpropagation. The gradient of this function with respect to any learnable parameter (e.g., *α_c_*, *P_c_*, *N_c_*) is used to update the fuzzy activation unit parameters during training.

One key advantage of the proposed SIT2-FRU activation structure is its low parameter count. Each fuzzy unit requires only three learnable parameters: *α_c_*, *P_c_*, and *N_c_*, representing the uncertainty width and the positive/negative slope parameters, respectively. Therefore, for a layer with *C* hidden units, the total number of fuzzy-related parameters is limited to 3 × *C*, which contributes to model efficiency and reduced overfitting risk. This quantity is significantly lower than the number of weights in deep learning networks, resulting in a more rapid convergence of the deep network to the target value. The previously mentioned benefits of type 2 fuzzy sets have resulted in their application alongside deep learning networks in this study to differentiate between healthy individuals and stroke patients under sensory-stimulated conditions [34]. We employ a SIT2-FRU as our activation function, which introduces fuzzy logic-based adaptability into the network structure.

As we have seen, machine learning and its subset, artificial intelligence, have been used optimally in various applications in recent years. These applications include vibrations [35], deep learning networks [36,37,38], COVID-19 [39], aerospace [40], financial markets [41,42,43,44,45,46,47], management and leadership [48,49], gaming and entertainment [50], materials and metallurgy [51], etc. In this research, we have also used machine learning algorithms to automatically diagnose brain tumors without medical intervention. Furthermore, recent studies have emphasized the applicability of AI-driven approaches in diverse healthcare contexts, such as brain and lung tumor detection [52,53], medical diagnostics and patient care [54,55], health-related text mining [56], computational modeling [57], supply chain optimization [58], manufacturing [59], and mental health analysis [60]. These findings reinforce the relevance and adaptability of our AI-based methodology in clinical evaluation and postural disorder classification. Additionally, other investigations have highlighted AI and machine learning capabilities in areas such as decentralized multi-agent learning [60], generative AI evolution [61], geotechnical engineering [62], technology-assisted language education [63], game-based vocabulary learning [64], FinTech transformation [65], financial technology adoption [66], architectural image classification [67], and AI applications in urban design [68].

The hybrid design combining CNNs with type-2 fuzzy logic was selected to address the uncertainty and variability inherent in postural control data under altered sensory conditions. CNNs efficiently extract local temporal features, while type-2 fuzzy activation improves robustness to noise and nonlinear dynamics. This architecture thus enables precise classification even in noisy or uncertain environments.

## 4. Proposed Method

The proposed methodology comprises multiple steps, which will be elucidated in detail below.

The first stage is identifying the traits that are used to predict sensory impairment. Various factors have been employed in the research on sensory dysfunction based on the data and assessment of the state control system of stroke patients and healthy persons under stimulated sensory situations.

To ascertain the condition of stroke patients, the second phase involved extracting and gathering the data that was accessible. A questionnaire to evaluate stroke patients’ state control systems and a laboratory study employing the EquiTest device to record the time series of postural fluctuations in various states of the sensory organization test were used to gather the available data for this purpose.

The collected data underwent normalization in the third phase. The data are therefore improved and ready for the following processes, which include model learning. Normalization makes it simpler to deal with by ensuring that each data set differs somewhat from the others and preventing large-scale data from distorting the conclusions. Consequently, their net input should fall within the sigmoid function’s range (between 0 and 1) in order to avoid premature neuronal saturation and balance the network’s data value. This keeps the weights from being too tiny and stops neurons from becoming saturated too soon. As a result, data standardization between 0 and 1 has been carried out in the suggested model.

The fourth stage presents the suggested network design, which combines type 2 fuzzy sets and convolutional networks. There are ten standard layers in the suggested architecture. The layers are arranged as follows:

(a) A convolutional layer that combines a batch normalizer layer, a maximum merging layer, a dropout layer, and a Leakey-ReLU function.
(b) In the preceding stage, the architecture is replicated nine more times.
(c) Two 2D matrices are linked to the outcomes of the last iteration.
(d) Each output is categorized and given a score using two completely linked layers.

**Figure 2:**
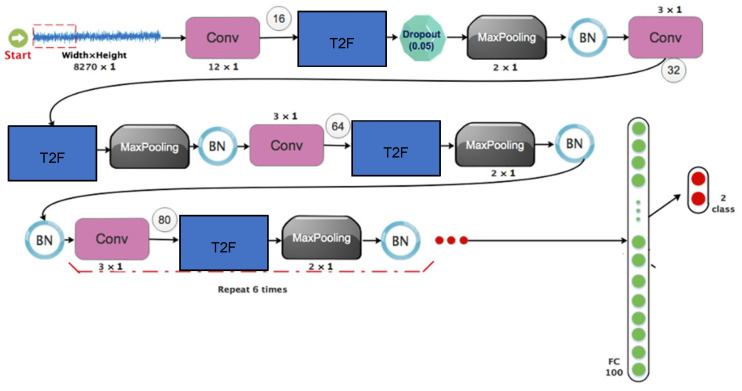
Suggested architecture for detecting strokes.

Figure 2 provides a visual representation of the network architecture stated above.

The suggested design takes into account a second scenario to ascertain the beneficial benefits of type 2 fuzzy sets. In the suggested design, these type 2 fuzzy functions have taken the role of the Leakey-Release functions. Table 2 displays the sizes of the filters, stages, and other components in the aforementioned design. A two-dimensional convolution operation was implemented, consistent with the spatial structure of the CoP signal maps. A kernel size of 3 × 3 was selected after comparative analysis with 5 × 5 kernels. While larger kernels can capture more context, the 3 × 3 configuration achieved optimal balance between computational efficiency and classification accuracy. Moreover, 3 × 3 kernels are widely adopted in deep architectures due to their flexibility in stacking layers and preserving spatial granularity.

In the proposed network, each SIT2-FRU activation unit includes three learnable parameters: *α*, *P*. These are initialized for each neuron (or channel) in every layer. Given that the architecture contains a total of 672 fuzzy units across 10 convolutional layers, the total number of fuzzy-specific learnable parameters is 2016. This compact parametrization contributes to model efficiency and low overfitting risk.

**Figure 3:**
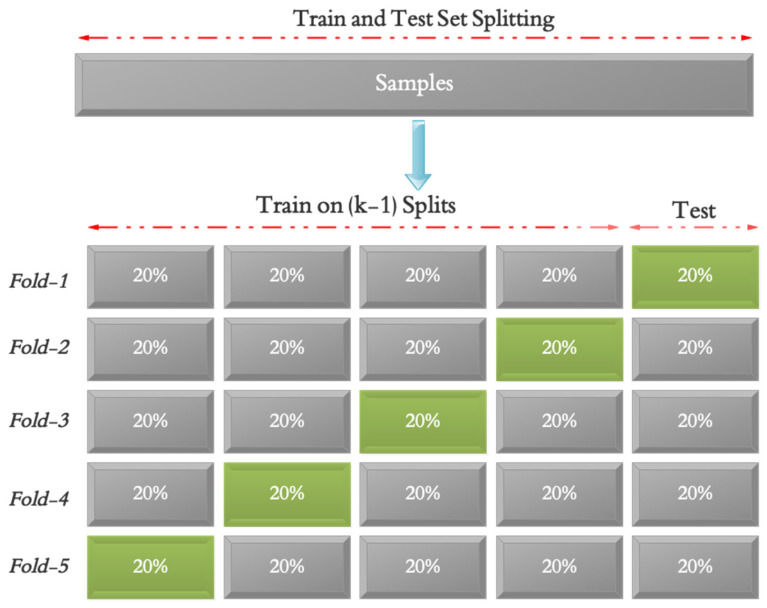
Five-fold cross validation process in the proposed model.

The data is used to determine the training and testing sets in the fifth phase of the suggested approach. According to earlier research, the training, testing, and validation sets are also set to contain 70% (5292 samples), 20% (2268 samples), and 10% (756 samples) of the data, respectively. The placement of data in the training and evaluation sets is also random. In addition, 5-fold cross validation was applied in the proposed model as shown in Figure 3 to ensure that overfitting does not occur in the network training process. Based on this assessment, all samples are included in the evaluation process. To address class imbalance, we used a class-weighted cross-entropy loss function, assigning higher weights to the minority class (stroke subjects), thereby encouraging the model to treat both classes equally during optimization.

The optimization of the network’s chosen parameters is established in the sixth and last stage of the suggested approach. In order for the network to have the least amount of error and converge to the intended value as quickly as possible, all of these parameters have been carefully chosen using the trial-and-error approach. In order to optimize the parameters and the cross-entropy algorithm for the error function with a training rate of 0.001 for the suggested deep network, the Adam optimizer was utilized. The suggested network’s optimum parameters are displayed in Table 3. Based on Table 3, the training process was conducted over 200 epochs for the baseline model and 60 epochs for the fuzzy-enhanced model. Adam optimizer with a learning rate of 0.001 and categorical cross-entropy loss was used. No data augmentation was applied, given the large size and diversity of the dataset. Overfitting was mitigated via dropout layers, batch normalization, and 5-fold cross-validation, which showed consistent performance across validation folds.

## 5. Results

This part will provide the suggested approach for Stroke detection, including data pre-processing, the proposed architecture, and the training and assessment set. The schematic overview of the proposed method is represented in Figure 2.

The study findings are examined both descriptively and analytically in this part. The simulation results are based on a machine running the Python programming language and the Cross library, with Corei9 specs and 32 RAM.

This section provides information on the several assessment indices used to diagnose stroke. The simulation results derived from the suggested model will next be examined. The suggested model has been assessed in this study using common metrics like sensitivity, accuracy, specificity, false positive rate, false negative rate, true negative rate, true positive rate, and ROC plot. As a result, the next section will cover how each of these indicators is calculated.

One common metric for assessing a classification test is sensitivity. Sensitivity may be expressed mathematically as the division of the true positive numbers by the total of the false negative and true positive numbers. The true negative numbers divided by the total of the true negative and false positive numbers is the specificity. The degree to which the measured number resembles the genuine number is known as accuracy. Another measure of the range of statistical dispersion is precision. The links between sensitivity, accuracy, specificity, and precision are as follows:

(11) Sensivity=Recall=TP/(FN+TP)

(12) Accuracy=(TP+TN)/(TP+TN+FP+FN)

(13) Specificity=Recall=TN/(TN+FP)

(14) Precision==TP/(FP+TP)

*TP* stands for the number of true positive samples, *TN* for the number of true negative samples, *FN* for the number of false negative samples, and *FP* for the number of false positive samples, in line with the previously described relations.

**Figure 4:**
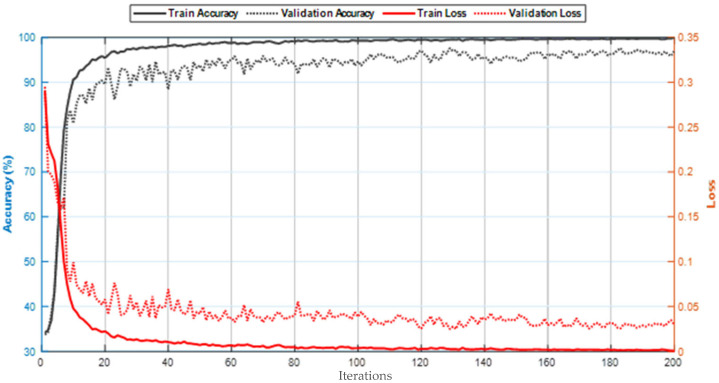
Accuracy and error criterion performance of the suggested model across 200 network iterations.

**Figure 5:**
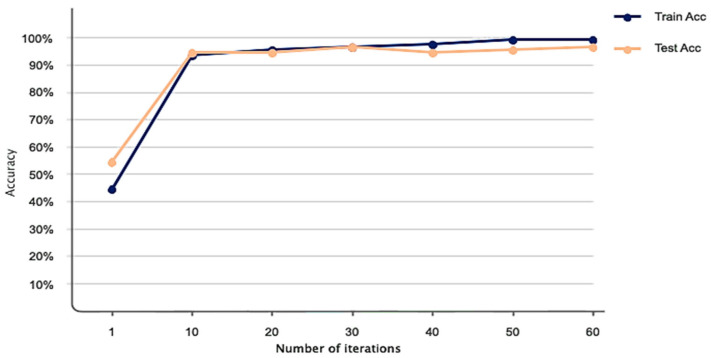
Accuracy criterion performance of the suggested model after 60 iterations in the second case.

**Figure 6:**
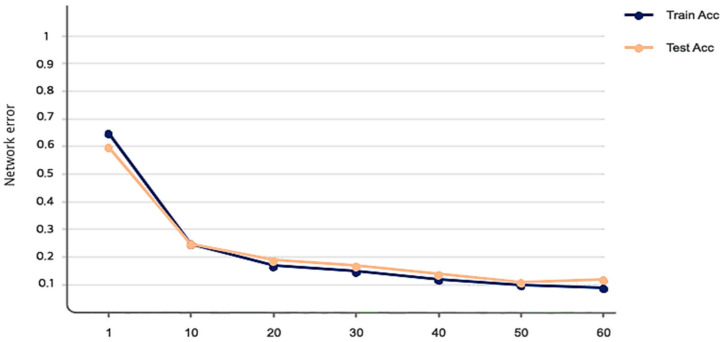
Network error measure performance of the suggested model after 60 iterations in the second case.

**Figure 7:**
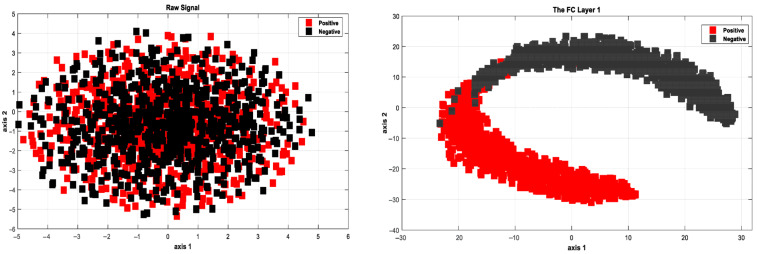
T-SNE plot of the distribution of class samples for the raw data (**left**) and the final layer of the network (**right**).

**Figure 8:**
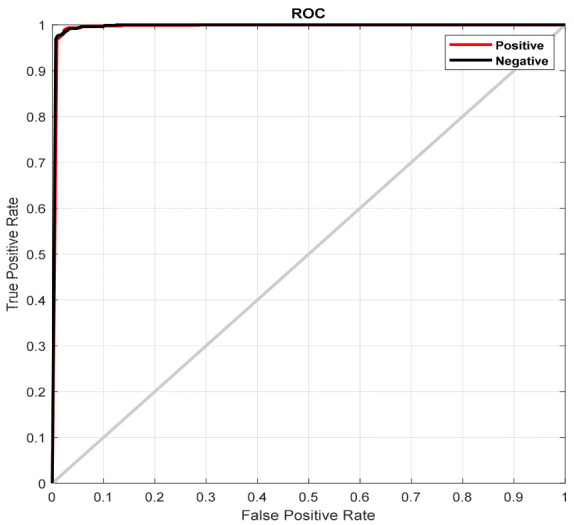
ROC curve statistical study for two distinct classes in the suggested model.

**Figure 9:**
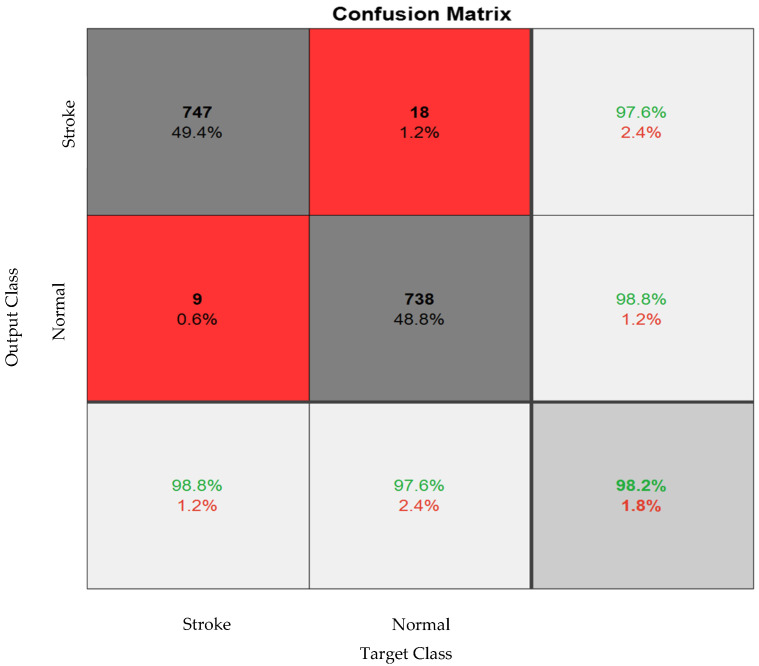
Confusion matrix for classifying two different classes (Gray color indicates correct sample detection, and red color indicates incorrect sample detection).

The simulation results of the proposed model based on the pertinent evaluation metrics will be provided in the following section. The suggested model’s accuracy and error in classifying the states of stroke patients and healthy individuals in simulated sensory environments are shown in Figure 4. At the 180th iteration, the error value in this figure has stabilized and reached equilibrium. The network error has dropped to around 0.35, which is its lowest figure. The same graphic shows that the final accuracy reaches 98%. The second case, where type 2 fuzzy sets are used as an alternative to the ReLU and Leaky ReLU functions, is presented in the architectural part of the proposed method. Based on 60 iterations for network training and verification, Figure 5 shows the accuracy results for the second scenario. With an accuracy of over 99%, the second recommended method shows great effectiveness in classifying stroke patients and healthy individuals in sensory-stimulated environments. Figure 6 shows the network error for the second situation across 60 trials. As more network iterations have been made, the error magnitude has decreased to its lowest value. The T-SNE diagram for the raw data and the last layer of the suggested network, which categorizes the condition of stroke patients and healthy individuals under sensory-stimulated circumstances, is shown in Figure 7. As can be seen, almost all of the samples in the last layer fall into one of two categories. The proposed effective design is responsible for this noteworthy difference. The statistical analysis of the ROC curve used to evaluate the effectiveness of the recommended design is shown in Figure 8. Each class’s two curves are clearly seen on the left side of the instrument, between 0.9 and 1. The confusion matrix is shown in Figure 9 and shows that only 9 samples out of 747 samples in the stroke class were incorrectly identified. Table 4 presents the results pertaining to the evaluation metrics, including accuracy, precision, specificity, and sensitivity. The model’s exceptional performance is shown by the table, which shows that all results obtained from different evaluation indices for stroke patients and healthy individuals in stimulated sensory conditions above 95%.

**Figure 10:**
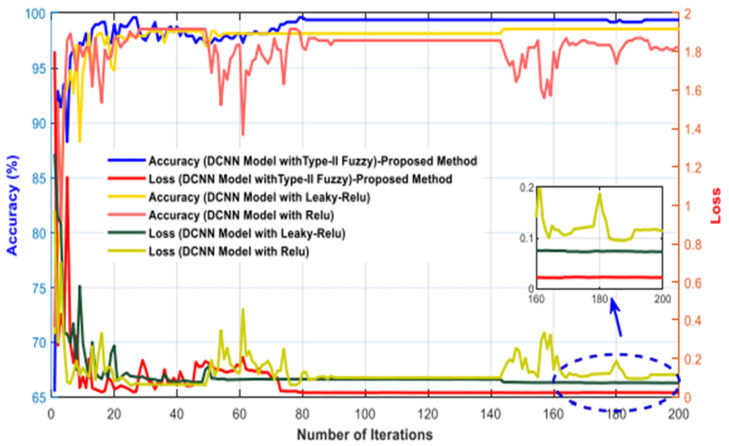
Evaluation of accuracy and error for two distinct situations with ReLU, Leakey-ReLU, and Fuzzy Type 2 functions.

**Figure 11:**
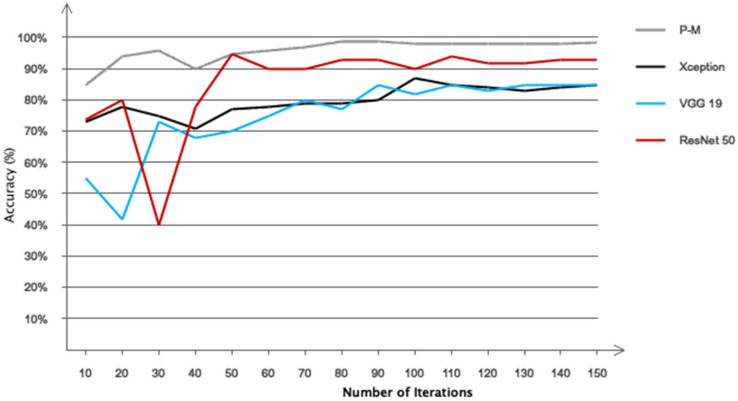
Comparison of the proposed model with pre-trained networks (P–M refers to the Proposed Method introduced in this study).

**Figure 12:**
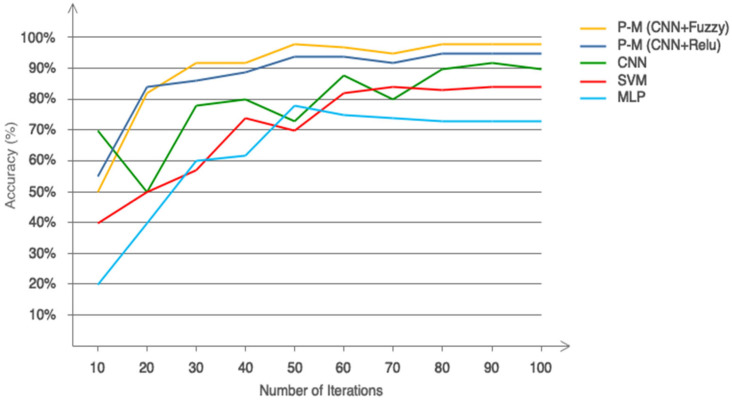
Performance comparison of the proposed model versus traditional classifiers.

**Figure 13:**
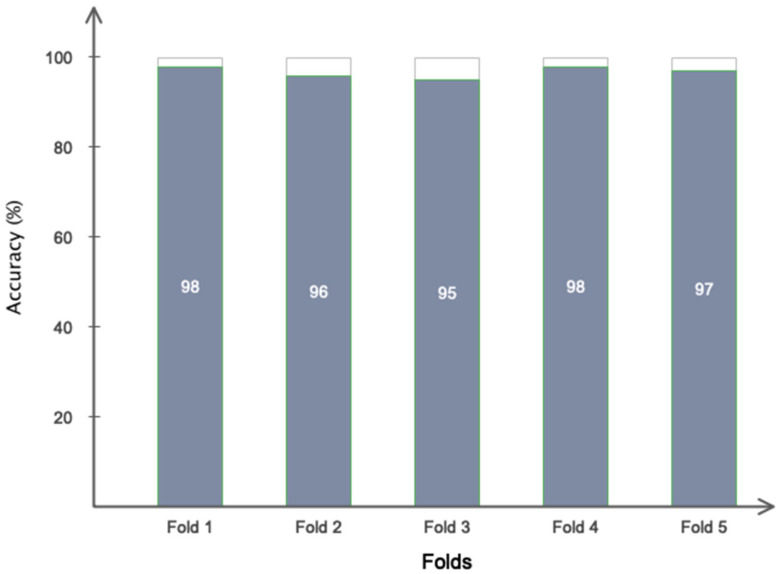
Evaluation of the proposed model with 5-fold cross-validation.

Figure 10, which is based on ReLU, Leaky ReLU, and Type 2 fuzzy sets, illustrates the performance of two distinct circumstances. As is obvious, Type 2 fuzzy sets have promising performance. Additionally, based on this number, it can be said that both scenarios’ performance offers an accuracy of more than 95% in identifying stroke patients and healthy people. Additionally, it is clear that the network exhibits greater convergence and less oscillation in the second situation with Type 2 fuzzy sets than in the first scenario with ReLU and Leaky ReLU activation functions. Table 5 also shows the computing efficiency of the two suggested situations. This table shows that the second scenario has a greater computing efficiency. Nonetheless, the second scenario’s ultimate classification accuracy for two distinct classes is greater. This table indicates that the first scenario’s computational efficiency is reduced when ReLU and Leaky ReLU activation functions are included. This table’s findings demonstrate that the first scenario may work well for real-time applications. Figure 11 shows the performance of the suggested model in 150 iterations using additional pre-trained networks, such as Xception, VGG19, and ResNet50. This figure shows that, when it comes to accuracy and convergence, the suggested model performs best when classifying two distinct classes. This chart demonstrates the effectiveness of type 2 fuzzy functions, which results in the suggested model having the fastest convergence and the least fluctuation. Although ResNet50 achieved competitive performance in our benchmarking experiment, it is primarily designed for image-based tasks and lacks mechanisms to handle signal uncertainty and semantic ambiguity in postural data. Our proposed model, which combines CNN with Type-2 fuzzy inference, explicitly addresses these challenges, making it more suitable for clinical applications involving noisy or variable physiological signals. Furthermore, the suggested model has been contrasted with other traditional methods, such as the support vector machine, multilayer perceptron, and simple CNN. The first two of these techniques rely on manual categorization and feature selection/extraction. Consequently, 29 separate performance characteristics were taken out of the data and categorized using the comparing classifiers. Figure 12 displays the simulation results after 100 network iterations. As can be observed, the second scenario has the best convergence and performance, and it has required the fewest iterations to achieve high accuracy in class distinction. Despite their poor computing efficiency, traditional approaches do not ensure that the feature vector for the classifier is optimum. The settings for the compared models are presented in Table 6. Figure 13 shows the results obtained according to the 5-fold cross validation. Based on the same figure, as can be seen, the results of the proposed model in different folds are almost equal and it can be concluded that the overfitting phenomenon did not occur in the network training process. Among the advantages of using type 2 fuzzy sets in this study are better uncertainty management, greater flexibility in modeling real-world complexities, improved performance in intelligent control systems, increased robustness against noise and environmental changes, and improved learning and inference in artificial intelligence-based systems compared to other methods.

**Figure 14:**
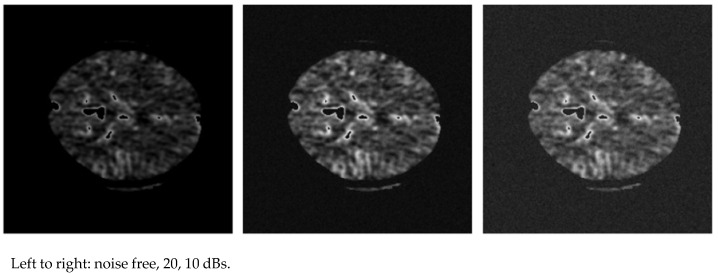

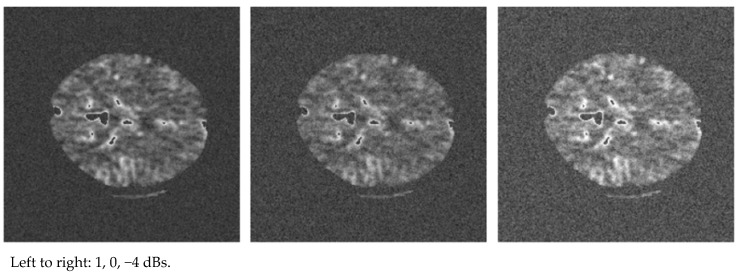
Proposed network resiliency in noisy settings in two situations.

**Figure 15:**
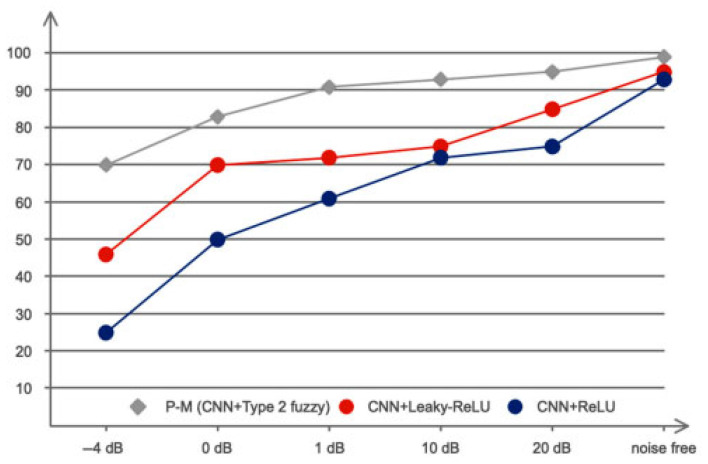
Comparison of classification accuracy under different noise levels for three models: P-M (CNN + Type-2 fuzzy), CNN + Leaky-ReLU, and CNN + ReLU.

White Gaussian noise has been intentionally introduced into the data at a variety of SNRs to assess the effectiveness of the suggested models in noisy settings. The noise added to a sample of images is shown in Figure 14. Figure 15, which shows the performance of the circumstances under consideration. This figure suggests that type 2 fuzzy functions are highly effective when dealing with a variety of uncertainties, including measurement noise, as seen by the 90% classification accuracy at 1 dB.

The model demonstrated strong generalization across a heterogeneous population of stroke patients and healthy individuals. Although detailed clinical stroke severity scores were not included, subject diversity in terms of age and postural control performance was inherently represented through the EquiTest-derived dataset. The proposed model maintained consistent accuracy across multiple cross-validation folds and demonstrated resilience to sensor noise, maintaining high classification performance even under low SNR conditions. These results suggest that the model is robust against inter-subject variability and may generalize well across different populations.

## 6. Discussion and Conclusions

This work aimed to develop an automated system using artificial intelligence approaches to recognize and diagnose the states of healthy persons and stroke patients under sensory-stimulated environments, given the global significance of stroke, particularly in Iran. Subsequently, after data preparation, deep convolutional networks were used in conjunction with type 2 fuzzy sets. The findings indicated that deep learning methodologies, owing to their superior reliability in feature selection and extraction, can accurately discern the health condition of people, distinguishing between healthy and ill subjects with high precision. The diagnosis and categorization of stroke patients in sensory-stimulated circumstances, based on the evaluation of sensory impairment according to the suggested model, achieved accuracy, precision, sensitivity, and specificity rates of 97%, 96%, 97%, and 96%, respectively. The suggested model was assessed in noisy conditions and showed good performance. Due to the outstanding performance of the suggested model, it is suitable for online applications. The compact nature of the proposed CNN-fuzzy model, with relatively low computational complexity, enables potential integration into clinical diagnostic systems and wearable devices. Its low number of trainable parameters and rapid convergence suggest feasibility for real-time inference. The model could be embedded into portable platforms such as balance-sensing insoles, rehabilitation robots, or smart posturography systems. Future efforts will focus on hardware acceleration and deployment on edge devices (e.g., NVIDIA Jetson, Raspberry Pi), with specific attention to minimizing latency and memory consumption to meet clinical standards.

This research, like other works, also has some shortcomings. Among these shortcomings is the consideration of binary classes for classification. It is suggested that, in future research, a large multi-class database be used for training and classification. It is also recommended that the performance of adversarial networks be evaluated to produce a larger database. Although our model achieves high classification performance on the present dataset, it was trained and tested within a single-center dataset collected under controlled experimental conditions. Future work will focus on evaluating the model on external datasets from different populations, clinical environments, and sensor configurations to ensure its generalizability and clinical robustness. Moreover, domain adaptation strategies may be explored to improve cross-site performance. While the current study focused on optimizing classification performance, we recognize that model interpretability is critical in clinical contexts. Future work will integrate explainability techniques such as SHAP or Integrated Gradients to determine which postural patterns or sensory conditions contribute most to the classification of stroke-related impairments. Such insights can enhance clinical trust and support data-driven therapy personalization.

The proposed deep learning-based model, through accurate classification of postural control impairments under altered sensory conditions, offers significant clinical benefits. It can serve as a non-invasive tool for early identification of balance disorders in stroke patients, enabling personalized rehabilitation strategies and supporting clinicians in therapy adjustment and monitoring.

## Figures and Tables

**Table 1:** Details of the filters and layers that are utilized in the suggested model.

Studies	Method
Chiari et al. [13]	Dynamic indices of postural control
Norris et al. [14]	Posturographic indices
Li et al. [15]	Postural control
Kiwanara et al. [16]	Postural control based on dissociated sensory systems
Richmond et al. [17]	CSP microstructural integrity
Da Oliveira et al. [18]	Core stability and sensorimotor training
Plereti et al. [19]	Machine learning
Tuncer et al. [20]	Postural control
Xu et al. [21]	Posture utilizing
Cherstvy et al. [22]	Diffusion processes with exponential and logarithmic time-dependent diffusion coefficients.
Novikov et al. [23]	MRS in cancer diagnosis and management

**Table 2:** Details of the filters and layers that are utilized in the suggested model.

Layer Number	Layer Type	Size and Filter Steps	Number of Filters	Output Value	Padding	Activation Function
1	Convolution1	12 × 1/8 × 1	16	1034 × 16	yes	Type II Fuzzy
2	Pooling1	2 × 1/2 × 1	16	517 × 16	no	
3	Convolution2	3 × 1/1 × 1	32	517 × 32	yes	Type II Fuzzy
4	Pooling2	2 × 1/2 × 1	32	258 × 32	no	
5	Convolution3	3 × 1/1 × 1	64	258 × 64	yes	Type II Fuzzy
6	Pooling3	2 × 1/2 × 1	64	129 × 64	no	
7	Convolution4	3 × 1/1 × 1	80	129 × 80	yes	Type II Fuzzy
8	Pooling4	2 × 1/2 × 1	80	64 × 80	no	
9	Convolution5	3 × 1/1 × 1	80	64 × 80	yes	Type II Fuzzy
10	Pooling5	2 × 1/2 × 1	80	32 × 80	no	
11	Convolution6	3 × 1/1 × 1	80	32 × 80	yes	Type II Fuzzy
12	Pooling6	2 × 1/2 × 1	80	16 × 80	no	
13	Convolution7	3 × 1/1 × 1	80	16 × 80	yes	Type II Fuzzy
14	Pooling7	2 × 1/2 × 1	80	8 × 80	no	
15	Convolution8	3 × 1/1 × 1	80	8 × 80	Yes	Type II Fuzzy
16	Pooling8	2 × 1/2 × 1	80	4 × 80	no	
17	Convolution9	3 × 1/1 × 1	80	8 × 80	yes	Type II Fuzzy
18	Pooling9	2 × 1/2 × 1	80	2 × 80	no	
19	Convolution10	3 × 1/1 × 1	80	2 × 80	yes	Type II Fuzzy
20	Pooling10	2 × 1/2 × 1	80	1 × 80	no	
21	Fully-connected	-	100	100	-	
22	Softmax	-	1	2	-	

**Table 3:** Details of the filters and layers that are utilized in the suggested model.

Parameters	Search Space	Optimal Value
Optimizer	RMSProp, Adam, Sgd, Adamax, Adadelta	Adam
Cost function	MSE, Cross-entropy	Cross-Entropy
Number of convolution layers	3, 5, 6, 10, 15	10
Filters in the first convolution layer	16, 32, 64, 128	16
Filters in the second convolution layer	16, 32, 64, 128	32
Filters in another convolution layer	16, 32, 64, 128	32
Size of filter in the first convolution layer	3, 16, 32, 64, 128	128
Size of filter in another convolution layers	3, 16, 32, 64, 128	16
Size of filter before the first convolution layer	0.2, 0.3, 0.4, 0.5	0.4
Dropout rate after the first convolution layer	0.2, 0.3, 0.4, 0.5	0.4
Batch size	4, 8, 10, 16, 32, 64	16
Learning rate	0.01, 0.001, 0.0001	0.001

**Table 4:** The suggested model’s performance in terms of precision, specificity, accuracy, and sensitivity.

Performance Metric	Stroke (%)	Normal (%)
Sensitivity	97.22	97.4
Accuracy	97.81	96.3
Specificity	96.66	97.7
Precision	96.65	97.7

**Table 5:** Comparison of the suggested model’s computing effectiveness across several functions.

Function	Stroke	Normal
ReLU	10,100	333
Leaky ReLU	11,542	421
Fuzzy sets	12,101	900

**Table 6:** Summary of Alternative Model Settings.

Model	Architecture/Type	Key Parameters	Input Features
SVM	RBF kernel	C = 1.0, gamma = ‘scale’	29 handcrafted
MLP	1 hidden layer	100 neurons, ReLU, Adam, LR = 0.001	29 handcrafted
CNN	2 Conv + Dense	Filters = 32/64, ReLU, 100 dense	Time series raw
VGG19	Pre-trained (fine-tuned)	LR = 1 × 10^−4^, top layers modified	Resized image format
ResNet50	Pre-trained (fine-tuned)	10 trainable layers, Adam	Resized image format
Xception	Pre-trained (fine-tuned)	Dropout = 0.5, Adam, batch = 32	Resized image format

## Data Availability

The raw data supporting the conclusions of this article will be made available by the authors on request.

## Abbreviations

Symbol	Definition
*X*	Input matrix or multichannel time-series data of CoP signals
*X*(*i*,*j*)	Element of the input matrix at row *i* and column *j*
*y*	Output label (binary classification: 0 = healthy, 1 = impaired)
*ŷᵢ*	Predicted class label for sample *i*
*K*	Convolution kernel (filter)—a small matrix of learnable weights (e.g., 3 × 3)
*K*(*m*,*n*)	Element of the convolution kernel at the *m*-th row and *n*-th column
*S*(*i*,*j*)	Value in the feature map at position (*i*,*j*), representing activation of a learned feature
*θ*	Trainable parameters of the ANN model
*pᵢ*	Probability assigned by the model to class *i*
*μ_A_*(*x*)	Membership function value of element *x* in fuzzy set A (Type-1)
*Ã*	Type-2 fuzzy set
*μ_Ã_*(*x*,*u*)	Membership function of a Type-2 fuzzy set, where *u* is the secondary grade
*J*	Objective or cost function used for training the model
*E*	Error term (e.g., classification error)
*L*	Loss function applied during optimization
*T*	Number of time steps in the time-series input
*C*	Number of channels in the input data
*N*	Number of samples in the dataset
*f_θ_*	ANN model parameterized by *θ*
⊗	Convolution operation

## References

[1] Arienti C., Lazzarini S.G., Pollock A., Negrini S. (2019). Rehabilitation interventions for improving balance following stroke: An overview of systematic reviews. PLoS ONE.

[2] Seyedkhamoushi F., Sassani Asl M., Seyedkhamooshi R., Abdollahi H.R., Sharifnia M. (2025). Multimodal Smart Eyeglasses for Adaptive Vision, Predictive Ocular and Hemodynamic Monitoring, and Emergency Response. World Intellectual Property Organization Patent.

[3] Blaszczyk J.W., Prince F., Raiche M., Hébert R. (2000). Effect of ageing and vision on limb load asymmetry during quiet stance. J. Biomech..

[4] Ghnemat R., Khalil A., Abu Al-Haija Q. (2023). Ischemic stroke lesion segmentation using mutation model and generative adversarial network. Electronics.

[5] Soltanpour M., Greiner R., Boulanger P., Buck B. (2021). Improvement of automatic ischemic stroke lesion segmentation in CT perfusion maps using a learned deep neural network. Comput. Biol. Med..

[6] Sadura-Sieklucka T., Czerwosz L.T., Kądalska E., Kożuchowski M., Księżopolska-Orłowska K., Targowski T. (2023). Is balance training using biofeedback effective in the prophylaxis of falls in women over the age of 65?. Brain Sci..

[7] Talis V., Kazennikov O. (2019). Effects of body turn on postural sway during symmetrical and asymmetrical standing. Exp. Brain Res..

[8] Vouriot A., Gauchard G.C., Chau N., Benamghar L., Lepori M.-L., Mur J.-M., Perrin P.P. (2004). Sensorial organisation favouring higher visual contribution is a risk factor of falls in an occupational setting. Neurosci. Res..

[9] Gray R., Kouhy R., Lavers S. (1995). Corporate social and environmental reporting: A review of the literature and a longitudinal study of UK disclosure. Account. Audit. Account. J..

[10] Kim S., Moon J. (2022). The effect of visual biofeedback balance training on time to stabilization and kinetic variables in patients with chronic ankle instability. Korean J. Sport Sci..

[11] Chamberlin C., Marmelat V., Rosen A.B., Burcal C.J. (2021). The effects of visual biofeedback and visual biofeedback scale size on single limb balance. J. Bodyw. Mov. Ther..

[12] Nichols D.S. (2002). Balance retraining after stroke using force platform biofeedback. Phys. Ther..

[13] Chiari L., Rocchi L., Cappello A. (2002). Stabilometric parameters are affected by anthropometry and foot placement. Clin. Biomech..

[14] Norris J.A., Marsh A.P., Smith I.J., Kohut R.I., Miller M.E. (2004). Ability of static and statistical mechanics posturographic measures to distinguish between age and fall risk. J. Biomech..

[15] Li L., Zhang S., Dobson J. (2019). The contribution of small and large sensory afferents to postural control in patients with peripheral neuropathy. J. Sport Health Sci..

[16] Keyvanara M., Sadigh M.J., Meijer K., Esfahanian M. (2021). A model of human postural control inspired by separated human sensory systems. Biocybern. Biomed. Eng..

[17] Richmond S.B., Whittier T.T., Peterson D.S., Fling B.W. (2021). Advanced characterization of static postural control dysfunction in persons with multiple sclerosis and associated neural mechanisms. Gait Posture.

[18] De Oliveira M.R., Fabrin L.F., de Oliveira Gil A.W., Benassi G.H., Camargo M.Z., da Silva R.A., de Lima R.R. (2021). Acute effect of core stability and sensory-motor exercises on postural control during sitting and standing positions in young adults. J. Bodyw. Mov. Ther..

[19] Tuncer D., Gurses H.N., Senaran H., Uzer G., Tuncay I. (2022). Evaluation of postural control in children with increased femoral anteversion. Gait Posture.

[20] Seifi N., Ghoodjani E., Majd S.S., Maleki A., Khamoushi S. (2025). Evaluation and Prioritization of Artificial Intelligence Integrated Block Chain Factors in Healthcare Supply Chain: A Hybrid Decision Making Approach. Comput. Decis. Mak. Int. J..

[21] Xu Y., Zeng Z., Oliveria C., Munoz R., de Almeida R., Quezada A., de Albuquerque V.H.C. (2022). Postural evaluation based on body movement and mapping sensors. Measurement.

[22] Novikov D.S., Fieremans E., Jespersen S.N., Kiselev V.G. (2019). Quantifying Brain Microstructure with Diffusion MRI: Theory and Parameter Estimation. NMR Biomed..

[23] Cherstvy A.G., Safdari H., Metzler R. (2021). Anomalous Diffusion, Nonergodicity, and Ageing for Exponentially and Logarithmically Time-Dependent Diffusivity: Striking Differences for Massive versus Massless Particles. J. Phys. D Appl. Phys..

[24] Gaurav K., Roy B., Bharti J. (2022). A Hybrid Deep Learning Model for Human Activity Recognition Using Wearable Sensors. Machine Intelligence and Smart Systems.

[25] Ahmadkhan K., Ahmadirad Z., Karaminezhad K., SeyedKhamoushi F., Karimi K., Khakpash F. (2025). A novel block-chain-based approach for enhanced food supply chain traceability and waste mitigation. Br. Food J..

[26] Sheykhivand S., Rezaii T.Y., Saatlo A.N., Romooz N. (2017). Comparison between different methods of feature extraction in BCI systems based on SSVEP. Int. J. Ind. Math..

[27] Basirat S., Raoufi S., Bazmandeh D., Khamoushi S., Entezami M. (2025). Ranking of AI-Based Criteria in Health Tourism Using Fuzzy SWARA Method. Comput. Decis. Mak. Int. J..

[28] Sabahi K., Sheykhivand S., Mousavi Z., Rajabioun M. (2023). Recognition COVID-19 cases using deep type-2 fuzzy neural networks based on chest X-ray image. Comput. Intell. Electr. Eng..

[29] Phan H., Andreotti F., Cooray N., Chén O.Y., De Vos M. (2018). Joint Classification and Prediction CNN Framework for Automatic Sleep Stage Classification. IEEE Trans. Biomed. Eng..

[30] Sheykhivand S., Yousefi Rezaii T., Mousavi Z., Meshini S. (2018). Automatic stage scoring of single-channel sleep EEG using CEEMD of genetic algorithm and neural network. Comput. Intell. Electr. Eng..

[31] Lin M.H., Sassani M., Golchin N., Jabbari Y., Boymatova Z., Rustambekovich J.U., Ugli Y.J.E., Atajanova S., Turdiyeva Y. (2025). Optimal Planning and Operation of the Smart Electrical Distribution Network Considering Stochastic Optimization Modeling and Energy Storage Systems. Oper. Res. Forum.

[32] Goodfellow I., Bengio Y., Courville A. (2016). Deep Learning.

[33] Beke A., Kumbasar T. (2019). Learning with Type-2 Fuzzy Activation Functions To Improve the Performance of Deep Neural Networks. Eng. Appl. Artif. Intell..

[34] Shen T., Wang J., Gou C., Wang F.-Y. (2020). Hierarchical fused model with deep learning and type-2 fuzzy learning for breast cancer diagnosis. IEEE Trans. Fuzzy Syst..

[35] Afsharfard A., Jafari A., Rad Y.A., Tehrani H., Kim K.C. (2023). Modifying Vibratory Behavior of the Car Seat to Decrease the Neck Injury. J. Vib. Eng. Technol..

[36] Khatami S.S., Shoeibi M., Salehi R., Kaveh M. (2025). Energy-Efficient and Secure Double RIS-Aided Wireless Sensor Networks: A QoS-Aware Fuzzy Deep Reinforcement Learning Approach. J. Sens. Actuator Netw..

[37] Mohammadabadi S.M.S., Zawad S., Yan F., Yang L. (2024). Speed Up Federated Learning in Heterogeneous Environments: A Dynamic Tiering Approach. IEEE Internet Things J..

[38] Sadeghi S., Niu C. (2024). Augmenting Human Decision-Making in K-12 Education: The Role of Artificial Intelligence in Assisting the Recruitment and Retention of Teachers of Color for Enhanced Diversity and Inclusivity. Leadersh. Policy Sch..

[39] Mahdavimanshadi M., Anaraki M.G., Mowlai M., Ahmadirad Z. A Multistage Stochastic Optimization Model for Resilient Pharmaceutical Supply Chain in COVID-19 Pandemic Based on Patient Group Priority. Proceedings of the 2024 Systems and Information Engineering Design Symposium (SIEDS).

[40] Azadmanesh M., Roshanian J., Georgiev K., Todrov M., Hassanalian M. (2024). Synchronization of Angular Velocities of Chaotic Leader-Follower Satellites Using a Novel Integral Terminal Sliding Mode Controller. Aerosp. Sci. Technol..

[41] Ahmadirad Z. (2024). Evaluating the Influence of AI on Market Values in Finance: Distinguishing Between Authentic Growth and Speculative Hype. Int. J. Adv. Res. Humanit. Law.

[42] Sadeghi S., Marjani T., Hassani A., Moreno J. (2022). Development of Optimal Stock Portfolio Selection Model in the Tehran Stock Exchange by Employing Markowitz Mean-Semivariance Model. J. Financ. Issues.

[43] Ahmadirad Z. (2024). The Beneficial Role of Silicon Valley’s Technological Innovations and Venture Capital in Strengthening Global Financial Markets. Int. J. Mod. Achiev. Sci. Eng. Technol..

[44] Dokhanian S., Sodagartojgi A., Tehranian K., Ahmadirad Z., Moghaddam P.K., Mohsenibeigzadeh M. (2024). Exploring the Impact of Supply Chain Integration and Agility on Commodity Supply Chain Performance. World J. Adv. Res. Rev..

[45] Ahmadirad Z. (2024). The Effects of Bitcoin ETFs on Traditional Markets: A Focus on Liquidity, Volatility, and Investor Behavior. Curr. Opin..

[46] Ahmadirad Z. (2024). The Banking and Investment in the Future: Unveiling Opportunities and Research Necessities for Long-Term Growth. Int. J. Appl. Res. Manag. Econ. Account..

[47] Gudarzi Farahani Y., Mirarab Baygi S.A., Abbasi Nahoji M., Roshdieh N. (2026). Presenting the Early Warning Model of Financial Systemic Risk in Iran’s Financial Market Using the LSTM Model. Int. J. Financ. Manag. Account..

[48] Khorsandi H., Mohsenibeigzadeh M., Tashakkori A., Kazemi B., Khorashadi Moghaddam P., Ahmadirad Z. (2024). Driving Innovation in Education: The Role of Transformational Leadership and Knowledge Sharing Strategies. Curr. Opin..

[49] Nezhad K.K., Ahmadirad Z., Mohammadi A.T. (2024). The Dynamics of Modern Business: Integrating Research Findings into Practical Management.

[50] Nawaser K., Jafarkhani F., Khamoushi S., Yazdi A., Mohsenifard H., Gharleghi B. (2024). The Dark Side of Digitalization: A Visual Journey of Research through Digital Game Addiction and Mental Health. IEEE Eng. Manag. Rev..

[51] Motta de Castro E., Bozorgmehrian F., Carrola M., Koerner H., Samouei H., Asadi A. (2025). Sulfur-Driven Reactive Processing of Multiscale Graphene/Carbon Fiber-Polyether Ether Ketone (PEEK) Composites with Tailored Crystallinity and Enhanced Mechanical Performance. Compos. Part B Eng..

[52] Golkarieh A., Rezvani Boroujeni S., Kiashemshaki K., Deldadehasl M., Aghayarzadeh H., Ramezani A. (2025). Breakthroughs in Brain Tumor Detection: Leveraging Deep Learning and Transfer Learning for MRI-Based Classification. Comput. Decis. Mak. Int. J..

[53] Rezaeianjam M., Khabazian A., Khabazian T., Ghorbani F., Abbasi T., Asghari S., Heidari F., Shiri A., Naderi M. (2025). Efficacy of Ozone Therapy in Dentistry with Approach of Healing, Pain Management, and Therapeutic Outcomes: A Systematic Review of Clinical Trials. BMC Oral Health.

[54] Sajjadi Mohammadabadi S.M., Seyedkhamoushi F., Mostafavi M., Borhani Peikani M., Gupta M., Kumar R., Lu Z. (2024). Examination of AI’s Role in Diagnosis, Treatment, and Patient Care. Transforming Gender-Based Healthcare with AI and Machine Learning.

[55] Golkarieh A., Kiashemshaki K., Rezvani Boroujeni S., Sadi Isakan N. (2025). Advanced U-Net Architectures with CNN Backbones for Automated Lung Cancer Detection and Segmentation in Chest CT Images. arXiv.

[56] Entezami Z., Davis C.H., Entezami M. (2025). An AI-Assisted Topic Model of the Media Literacy Research Literature. Media Lit. Acad. Res..

[57] Mohaghegh A., Huang C. (2025). Feature-Guided Sampling Strategy for Adaptive Model Order Reduction of Convection-Dominated Problems. arXiv.

[58] Ahmadirad Z. (2025). The Role of AI and Machine Learning in Supply Chain Optimization. Int. J. Mod. Achiev. Sci. Eng. Technol..

[59] Dehghanpour Abyaneh M., Narimani P., Javadi M.S., Golabchi M., Attarsharghi S., Hadad M. (2024). Predicting Surface Roughness and Grinding Forces in UNS S34700 Steel Grinding: A Machine Learning and Genetic Algorithm Approach to Coolant Effects. Physchem.

[60] Mohammadabadi S.M.S., Yang L., Yan F., Zhang J. Communication-Efficient Training Workload Balancing for Decentralized Multi-Agent Learning. Proceedings of the 2024 IEEE 44th International Conference on Distributed Computing Systems (ICDCS).

[61] Sajjadi Mohammadabadi S.M. (2025). From Generative AI to Innovative AI: An Evolutionary Roadmap. arXiv.

[62] Barati-Nia A., Parrott A.E., Sorenson K., Moug D.M., Khosravifar A. (2025). Comparing Cyclic Direct Simple Shear Behavior of Fine-Grained Soil Prepared with SHANSEP or Recompression Approaches. Geotechnical Frontiers.

[63] Asadi M., Taheri R. (2024). Enhancing Peer Assessment and Engagement in Online IELTS Writing Course through a Teacher’s Multifaceted Approach and AI Integration. Technol. Assist. Lang. Educ..

[64] Abbasi E., Dwyer E. (2024). The Efficacy of Commercial Computer Games as Vocabulary Learning Tools for EFL Students: An Empirical Investigation. Sunshine State TESOL J..

[65] Pazouki S., Jamshidi M.B., Jalali M., Tafreshi A. (2025). Transformative Impact of AI and Digital Technologies on the FinTech Industry: A Comprehensive Review. Int. J. Adv. Res. Humanit. Law.

[66] Pazouki S., Jamshidi M.B., Jalali M., Tafreshi A. (2025). Artificial Intelligence and Digital Technologies in Finance: A Comprehensive Review. J. Econ. Financ. Account. Stud..

[67] Karkehabadi A., Sadeghmalakabadi S. Evaluating Deep Learning Models for Architectural Image Classification: A Case Study on the UC Davis Campus. Proceedings of the 2024 IEEE 8th International Conference on Information and Communication Technology (CICT).

[68] Naseri S. (2024). AI in Architecture and Urban Design and Planning: Case Studies on Three AI Applications. GSC Adv. Res. Rev..

